# Highly Stable Electronics Based on β‐Ga_2_O_3_ for Advanced Memory Applications

**DOI:** 10.1002/advs.202413846

**Published:** 2025-02-05

**Authors:** Xiaoxi Li, Yu‐Chun Li, Yingguo Yang, Bitao Dong, Yuhang Liu, Lina Li, Linfeng Pan, Gengsheng Chen, Yue Hao, Genquan Han

**Affiliations:** ^1^ Hangzhou Institute of Technology Xidian University Hangzhou 311200 China; ^2^ School of Microelectronics Xidian University Xi'an 710071 China; ^3^ State Key Laboratory of ASIC and System Shanghai Institute of Intelligent Electronics & Systems School of Microelectronics Fudan University Shanghai 200433 China; ^4^ Shanghai Synchrotron Radiation Facility (SSRF) Zhangjiang Lab Shanghai Advanced Research Institute Chinese Academy of Sciences Shanghai 201204 China; ^5^ State Key Laboratory of Photovoltaic Science and Technology Fudan University Shanghai 200433 China; ^6^ State Key Laboratory for Mechanical Behavior of Materials School of Materials Science and Engineering Xi'an Jiaotong University Xi'an 710049 China; ^7^ Department of Materials Sciences and Engineering Division of Solid State Physics Angstrom Laboratory Uppsala University Uppsala SE‐75105 Sweden; ^8^ Department of Chemical Engineering and Biotechnology University of Cambridge Cambridge CB2 1TN UK; ^9^ Frontiers Science Center for Transformative Molecules, School of Chemistry and Chemical Engineering, Zhangjiang Institute for Advanced Study Shanghai Jiao Tong University Shanghai 200240 China

**Keywords:** dynamic random‐access memory, stability, wide‐bandgap semiconductors, *β*‐Ga_2_O_3_/h‐BN heterostructure

## Abstract

Wide‐bandgap (WBG) semiconductors are at the forefront of driving innovations in electronic technology, perpetuating Moore's Law and opening up new avenues for electronic devices. Although *β*‐Ga_2_O_3_ has attracted extensive research interest in advanced electronics, its high‐temperature and high‐speed volatile memory applications in harsh environment has been largely overlooked. Herein, a high‐performance hexagonal boron nitride (h‐BN)/*β*‐Ga_2_O_3_ heterostructure junction field‐effect transistor (HJFET) is fabricated, exhibiting an off‐state current as low as ≈10 fA, a high on/off current ratio of ≈10^8^, a low contact resistance of 5.6 Ω·mm, and an impressive field‐effect electron mobility of 156 cm^2^  （Vs）^−1^. Notably, the current h‐BN/*β*‐Ga_2_O_3_ HJFET exhibits outstanding thermal reliability in the ultra‐wide temperature range from 223 to 573 K, as well as long‐term environmental stability in air, which confirms its inherent capability of operation in harsh environments. Moreover, the h‐BN/*β*‐Ga_2_O_3_ HJFET demonstrates successful applications for accelerator‐in‐memory computing fields, including dynamic random‐access memory structure and neural network computations. These superior characteristics position *β*‐Ga₂O₃‐based electronics as highly promising for applications in extreme environments, with particular relevance to the automotive, aerospace, and sensor sectors.

## Graphical abstract

The h‐BN/*β*‐Ga_2_O_3_ heterostructure junction field‐effect transistor with exceptional stability, featuring a high on/off current ratio of ~108 and an impressive field‐effect electron mobility of 156 cm^2^/Vs.

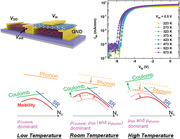

## 1 Introduction

It is increasingly recognized that silicon (Si)‐based transistors are approaching their projected scaling limits, so exploring new channel materials and device structures to adapt to new applications is imperative.^[^ ^1^, ^2^, ^3^ ^]^ Wide‐bandgap (WBG) materials such as Gallium Nitride, Silicon Carbide, and Gallium Oxide (Ga_2_O_3_) offer distinct advantages over silicon‐based devices, allowing for smaller, faster, more efficient, and more reliable electronics, especially in high‐power scenarios.^[^ ^4^, ^5^, ^6^ ^]^ WBG semiconductors allow devices to operate at much higher voltages, frequencies, and temperatures due to their outstanding material properties. In particular, WBG‐based electronic devices can operate at ambient temperatures over 300 °C without external cooling, unlike Si‐based devices, which suffer from severe reliability issues such as higher leakage currents and higher internal junction temperatures when operating above 200 °C.^[^ ^7^ ^]^ This inherent tolerance for high operating temperatures is a major advantage for WBG‐based electronic devices, making them ideal for critical applications in harsh environments, such as the automotive, aerospace, combustion systems, nuclear reactors, and energy production fields.^[^ ^8^, ^9^, ^10^ ^]^

Among these WBG materials, only *β*‐Ga_2_O_3_ single crystal can be homogeneously grown even in the early stages of its development.^[^ ^11^ ^]^ And *β*‐Ga_2_O_3_ with its unique material properties is considered as the most promising material for the next‐generation power electronics, solar‐blind ultraviolet photodetectors, and sensors.^[^ ^12^, ^13^ ^]^ *β*‐Ga_2_O_3_ has a large direct bandgap of 4.6–4.9 eV, an ultrahigh critical breakdown electrical field of 8 MV cm^−1^, a high saturation electron velocity of 2 × 10^7^ cm s^−1^, a high Baliga's figure‐of‐merit of 3244, an outstanding thermal and chemical stability.^[^ ^14^, ^15^ ^]^ In recent years, various high‐quality *β*‐Ga_2_O_3_ bulk single crystals have been grown by affordable edge‐defined film‐fed methods, the Czochralski method, the optical floating zone (OFZ) method, and so on.^[^ ^16^ ^]^ And numerous types of *β*‐Ga_2_O_3_‐based electronic devices have been fabricated and developed such as Schottky diode, p‐n diode, metal–insulator semiconductor field‐effect transistor, and heterostructure junction field‐effect transistor (HJFET).^[^ ^15^, ^17^, ^18^, ^19^ ^]^ Recent research aims to combine *β*‐Ga_2_O_3_ with other 2D materials to further improve the performance of *β*‐Ga_2_O_3_‐based devices. Tetzner^[^ ^20^ ^]^ et al. fabricated SnO/*β*‐Ga_2_O_3_ HJEFT which exhibits stable switching characteristics with a high on/off current ratio (*I*_on_/*I*_off_) of >10^6^, a specific on‐resistance below 4 mΩ·cm^2^, and a large breakdown voltage of 750 V. Kim^[^ ^21^ ^]^ et al. designed hexagonal boron nitride (h‐BN)/*β*‐Ga_2_O_3_ HJFET which displays a high *I*_on_/*I*_off_ of ≈10^7^ and a field‐effect mobility (*μ*_fe_) of 1.8 cm^2^ Vs^−1^. More Recently, Kim^[^ ^22^ ^]^ et al. demonstrated WSe_2_/*β*‐Ga_2_O_3_ HJFET which shows excellent transport properties with a low subthreshold swing (*SS*) of 76.8 mV dec.^−1^, a high *I*_on_/*I*_off_ of 6.6 × 10^7^, and a high *μ*_fe_ of 10.0 cm^2^ Vs^−1^. Despite the promising potential of *β*‐Ga_2_O_3_‐based HJFETs, there remains a critical gap in comprehensive studies addressing their reliability across high and low temperatures and their long‐term environmental stability. This presents a unique opportunity to innovate in high‐temperature memory technology. By focusing on the development of high‐speed volatile memory solutions specifically designed for harsh environments, our work aims to bridge this critical gap, paving the way for transformative advancements in memory applications.

In this work, a h‐BN/*β*‐Ga_2_O_3_ HJFET with outstanding electrical properties was fabricated which exhibits a low off‐state current (*I*_off_) of ≈10 fA, a high *I*_on_/*I*_off_ of ≈10^8^, a low contact resistance (*R*_c_) of 5.6 Ω·mm and a high μ_fe_ of 156 cm^2^  （Vs）^−1^. The temperature stability of h‐BN/*β*‐Ga_2_O_3_ HJFET in a temperature from 223 to 573 K and the long‐term stability in an air ambient has been systematically studied. Besides, a high‐performance logic inverter based on the h‐BN/*β*‐Ga_2_O_3_ HJFET is presented. As a proof‐of‐concept, the accelerator‐in‐memory computing function of *β*‐Ga_2_O_3_‐based on dynamic random‐access memory (DRAM) array and the three‐layer fully connected neural network have also been built and demonstrated. The results show that the *β*‐Ga_2_O_3_‐based device has great potential for harsh environment applications such as high‐temperature, high voltage, and high‐power fields.

## 2 Results and Discussion

**Figure 1:**
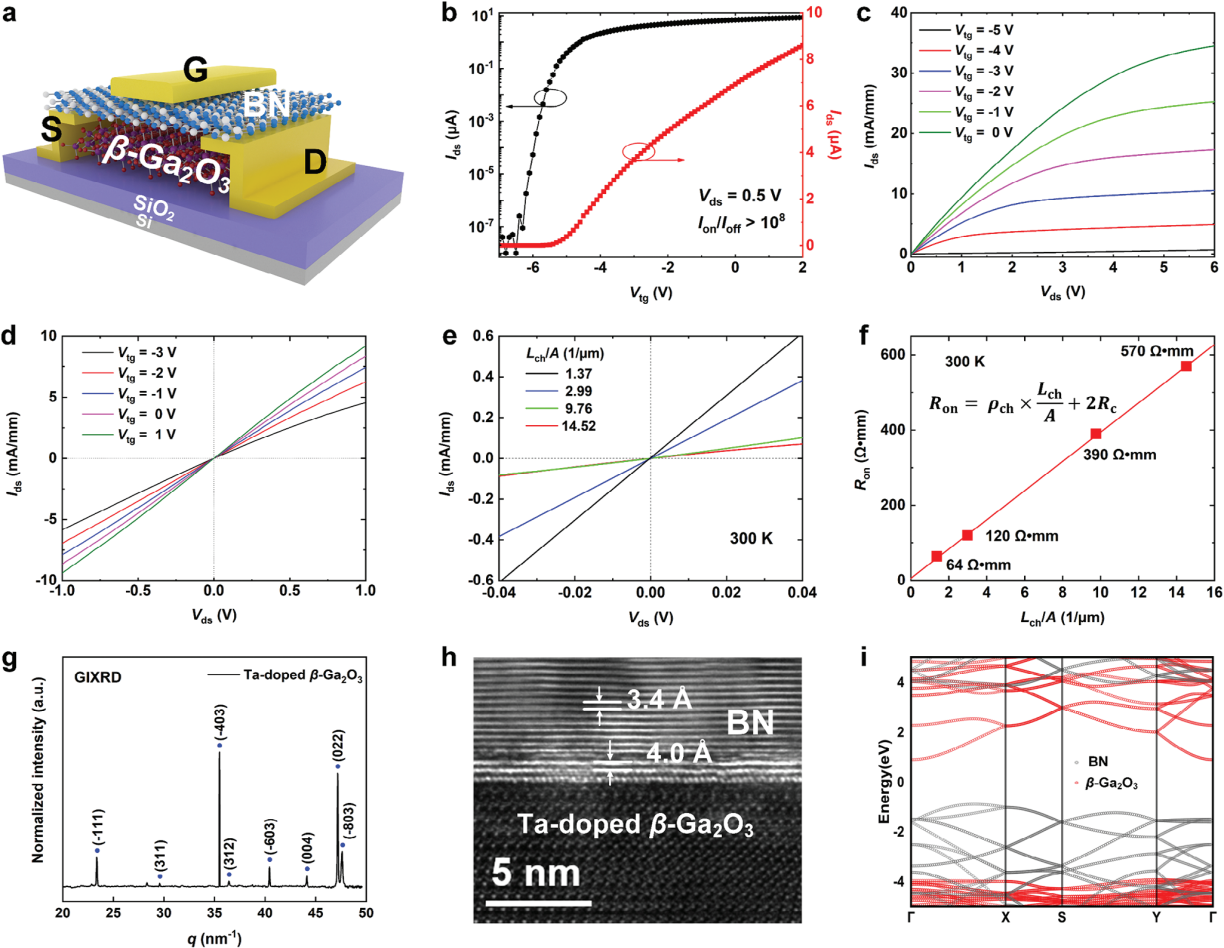
a) Schematic device structure of the h‐BN/β‐Ga_2_O_3_ HJFET. b) The transfer curves of the h‐BN/β‐Ga_2_O_3_ HJFET in the log‐ and linear‐ scales. c) The output curves of the h‐BN/β‐Ga_2_O_3_ HJFET. d) Ohmic contact characteristics of the device under different Vtg values. e) Ohmic contact characteristics of the h‐BN/β‐Ga_2_O_3_ HJFET with different channel lengths. f) The contact resistance of the h‐BN/β‐Ga_2_O_3_ HJFET. g) GIXRD spectrum of β‐Ga_2_O_3_ bulk single crystal. h) The HR‐TEM cross‐section image of the interface of h‐BN/β‐Ga_2_O_3_ heterostructure. i) The orbital‐resolved band structure of h‐BN/β‐Ga_2_O_3_ heterostructure.

**Figure** **1**a displays the schematic illustration of h‐BN/*β*‐Ga_2_O_3_ HJFET. 2D material h‐BN is used as the gate dielectric which has a wide bandgap of 5.97 eV, a high dielectric breakdown field (8–12 MV cm^−1^), and excellent thermal conductivity (1700–2000 W m K^−1^).^[^ ^23^, ^24^, ^25^ ^]^ The optical micrograph of the fabricated h‐BN/*β*‐Ga_2_O_3_ HJFET is shown in Figure S1 (Supporting Information). The length and width of the *β*‐Ga_2_O_3_ channel are ≈6.0 and ≈2.0 µm, respectively. The thicknesses of h‐BN dielectric layer and *β*‐Ga_2_O_3_ channel layer are measured to be ≈20 and ≈240 nm by atomic force microscopy (AFM) in Figure S2 (Supporting Information). Next, the electronic properties of h‐BN/*β*‐Ga_2_O_3_ HJFET were investigated. Figure 1b shows the transfer curves of h‐BN/*β*‐Ga_2_O_3_ HJFET in log and linear‐scales at *V*_ds_ = 0.5 V. It is observed that the h‐BN/*β*‐Ga_2_O_3_ HJFET exhibits excellent switching characteristics with a low *I*_off_ of ≈10 fA and a high *I*_on_/*I*_off_ of ≈10^8^, which is sufficient for the application of logic circuits.^[^ ^26^ ^]^ The low *I*_off_ of the fabricated device also benefits from the smooth and dangling bond‐free surface of the gate dielectric h‐BN.^[^ ^27^ ^]^ Furthermore, a steep *SS* of 87 mV dec^−1^ is extracted by using the equation $SS=\frac{dV_{\mathrm{tg}}}{d\log I_{\mathrm{ds}}}$, the steep *SS* could effectively reduce the power consumption in the WBG‐based complementary metal–oxide–semiconductor circuits.^[^ ^28^ ^]^ In addition, the *μ*_fe_ was also extracted from the following equation^[^ ^29^ ^]^

(1) $$\mu_{fe}=\frac{L}{W\times C_{ox}\times V_{ds}}\times g_{m}$$

where *L* and *W* are the length and width of the *β*‐Ga_2_O_3_ channel, *C*_ox_ is the unit‐area capacitance of gate dielectric h‐BN, *g*_m_ is the maximum value of transconductance of the h‐BN/*β*‐Ga_2_O_3_ HJFET. First, *C*_ox_ is calculated to be 1.77 × 10^−7^ F cm^−2^ by using the formula of $C_{ox}=\frac{\varepsilon_{0}\varepsilon_{r}}{d}$ where ε_0_ is the vacuum permittivity (8.854 × 10^−12^ F m^−1^), ε_*r*_ is the relative permittivity of h‐BN of 4, and *d* is the dielectric thickness of h‐BN (20 nm).^[^ ^24^ ^]^ The *g*_m_ as a function of *V*_tg_ is extracted by using the formula of $g_{m}=\frac{\partial I_{\mathrm{ds}}}{\partial V_{\mathrm{tg}}}$(Figure S3, Supporting Information). The transconductance first increases and then decreases as the gate voltage increases. Initially, as the gate voltage increases, the carrier concentration in the channel rises, leading to higher transconductance. However, at higher gate voltages, the electric field near the gate‐channel interface becomes stronger, which increases carrier scattering. This increased scattering reduces carrier mobility in the channel, leading to a decrease in transconductance.^[^ ^30^, ^31^, ^32^ ^]^ The *g*_m_ reaches at ≈2.31 mS mm^−1^ and the peak value of μ_fe_ is calculated to be 156 cm^2^ (Vs)^−1^ from Figure 1b. The high μ_fe_ can be attributed to the high‐quality interface between h‐BN and *β*‐Ga_2_O_3_, which effectively reduces charge carrier scattering. Additionally, h‐BN, as a dielectric layer, improves the overall interface quality by reducing defect states, thereby enhancing carrier injection and transport efficiency.^[^ ^33^ ^]^ The high μ_fe_ value illustrates that the fabricated h‐BN/*β*‐Ga_2_O_3_ HJFET is suitable for high‐speed integrated circuits and high‐speed random‐access memory applications.^[^ ^34^ ^]^ Besides, to comprehensively evaluate the mobility of the device, the split *C*‐*V* method is applied to extract the mobility which considers the trap density of the h‐BN/*β*‐Ga_2_O_3_ interface. The corresponding formula is as follows^[^ ^35^ ^]^

(2) $$\mu =\frac{L}{W}\frac{I_{ds}}{Q_{i}V_{ds}}$$

where *Q*_i_ is the inversion charge which is defined by $\int C_{\mathrm{gc}}dV_{\mathrm{tg}}$ where *C*_gc_ is gate‐channel capacitance. The measured *C_gc_‐V*_tg_ curves at 1, 10, 20, and 50 kHz are shown in Figure S4 (Supporting Information). The mobility extracted using this method is 91 cm^2^ (Vs)^−1^ which the *C_gc_‐V*_tg_ curve at 10 kHz was taken. Notably, the value of 91 cm^2^ (Vs)^−1^ stands as a strong competitor against the performance of existing *β*‐Ga₂O₃‐based devices.^[^ ^20^, ^21^, ^22^ ^]^ Figure 1c shows the output curves of h‐BN/*β*‐Ga_2_O_3_ HJFET where *V*_tg_ varied from −5 to 0 V with a step of 1 V. Remarkably, the *I*_ds_ is modulated effectively by *V*_tg_ with sharp pinch‐off and outstanding saturation properties. A high saturation current density of 34.5 mA mm^−1^ at *V*_tg_ = 0 V is observed. In addition, the resistance of the device is related with power dissipation and temperature elevation. Figure 1d shows the linear trend of the *I*_ds_‐*V*_ds_ curves under different *V*_tg_ values, indicating a perfect Ohmic contact behavior of h‐BN/*β*‐Ga_2_O_3_ HJFET. The value of on‐state resistance (*R*_on_) can be calculated to be 120 Ω·mm. But it is known that *R*_on_ is composed of *R*_c_ and channel resistance (*R*_ch_). In most cases, *R*_c_ at metal/semiconductor interface has a large impact on device performance.^[^ ^36^ ^]^ Therefore, the low *R*_c_ is expected to make h‐BN/*β*‐Ga_2_O_3_ HJFETs promising candidates for next‐generation high‐power and high‐voltage switching devices.^[^ ^37^ ^]^ Here, the transmission line measurements method is used to extract *R*_c_ by following the equation^[^ ^29^ ^]^

(3) $$R_{on}=\rho_{ch}\times \frac{L}{A}+2R_{c}$$

where *ρ*_ch_, *L*, and *A* are the *β*‐Ga_2_O_3_ channel resistivity, the channel length, and the cross‐sectional area, respectively. Figure 1e shows the Ohmic contact characteristics of h‐BN/*β*‐Ga_2_O_3_ HJFET with different channel lengths. According to the above formula, the functional relationship between *L*/*A* and *R*_on_ is plotted in Figure 1f. And 2*R*_c_ was extracted to be a small value of 5.6 Ω·mm, suggesting that a very low *R*_c_ of 2.8 Ω·mm can be realized by using the Ti/Al/Ni/Au stack electrodes. It can be explained that during the rapid thermal annealing (RTA) process in a high‐purity N_2_ ambient, the formation of the Ti‐Al inter‐metallic phase could serve as a reactive site that interacts with *β*‐Ga_2_O_3_, generating a significant number of oxygen vacancies at the interface. This may lead to the formation of TiO_x_ or AlO_x_ phases with distinct Fermi levels, potentially matching those of the oxygen vacancies in *β*‐Ga_2_O_3_, or it could enhance the chemical bonding at the interface, facilitating the formation of a good Ohmic contact.^[^ ^38^ ^]^

To reveal the origin of the enhancing electrical properties of the fabricated h‐BN/*β*‐Ga_2_O_3_ HJFET, the grazing incident X‐ray diffraction (GIXRD), the high‐resolution transmission electron microscopy (HR‐TEM), and Raman spectroscopy measurements are performed. Figure 1g displays the azimuthally integrated 1D GIXRD spectrum of *β*‐Ga_2_O_3_ bulk single crystal with the X‐ray wavelength of 0.6887 Å, which derived from the two‐dimensional (2D)‐GIXRD pattern (Figure S5, Supporting Information). The three typical diffraction peaks located at *q* = 23.5, 35.1, and 47.0 nm^−1^ match well with the monoclinic structure from the standard PDF card (no.76‐0573) and correspond to the (−111), (−403), and (022) crystal planes of *β*‐Ga_2_O_3_, respectively.^[^ ^39^ ^]^ The GIXRD spectrum clearly shows the excellent crystallization quality of *β*‐Ga_2_O_3_ bulk single crystal without other impurity phases. Next, the Raman spectrum of *β*‐Ga_2_O_3_ bulk single crystal is shown in Figure S6 (Supporting Information). Thirteen peaks were identified at 111, 114, 144, 169, 200, 320, 346, 416, 475, 631, 658, and 767 cm^−1^, corresponding to the A1, B1, B2, A2, A3, A4, A5, A6, B4, A8, A9, and A10 phonon modes, respectively. The high intensity and sharpness of these peaks align well with reported results, further confirming the high crystallinity of the *β*‐Ga_2_O_3_ single crystal sample.^[^ ^40^, ^41^ ^]^ Subsequently, the HR‐TEM cross‐section image of the interface h‐BN/*β*‐Ga_2_O_3_ heterostructure is shown in Figure 1h. Interestingly, the h‐BN/*β*‐Ga_2_O_3_ heterostructure has an ultraclean interface, demonstrating a good lattice match between *β*‐Ga_2_O_3_ layer and h‐BN layer.^[^ ^42^ ^]^ Furthermore, the corresponding energy band diagrams of *β*‐Ga_2_O_3_/h‐BN heterostructure were calculated utilizing density functional theory (DFT) in Figure 1i. More details of the DFT calculations are in Supporting Information. Visibly, the h‐BN/*β*‐Ga_2_O_3_ heterostructure formed the type‐II heterostructure and the bandgap of the h‐BN/*β*‐Ga_2_O_3_ heterostructure is less than that of a single material (h‐BN or *β*‐Ga_2_O_3_).^[^ ^43^ ^]^ When h‐BN and *β*‐Ga_2_O_3_ are integrated together, the h‐BN/*β*‐Ga_2_O_3_ heterostructure interface would form a space charge area because of the diffusion of carriers from high to low concentration and generate a built‐in electric field to maintain balance across the h‐BN/*β*‐Ga_2_O_3_ interface.^[^ ^44^ ^]^ The formation of a built‐in electric field at the *β*‐Ga_2_O_3_/h‐BN heterojunction facilitates the efficient separation and confinement of charge carriers, enhancing carrier mobility by reducing scattering. This improvement in mobility can lead to faster device performance. Notably, the combination of *β*‐Ga_2_O_3_ and h‐BN represents a novel approach in this field, warranting emphasis due to its potential to advance FET applications significantly.

**Figure 2:**
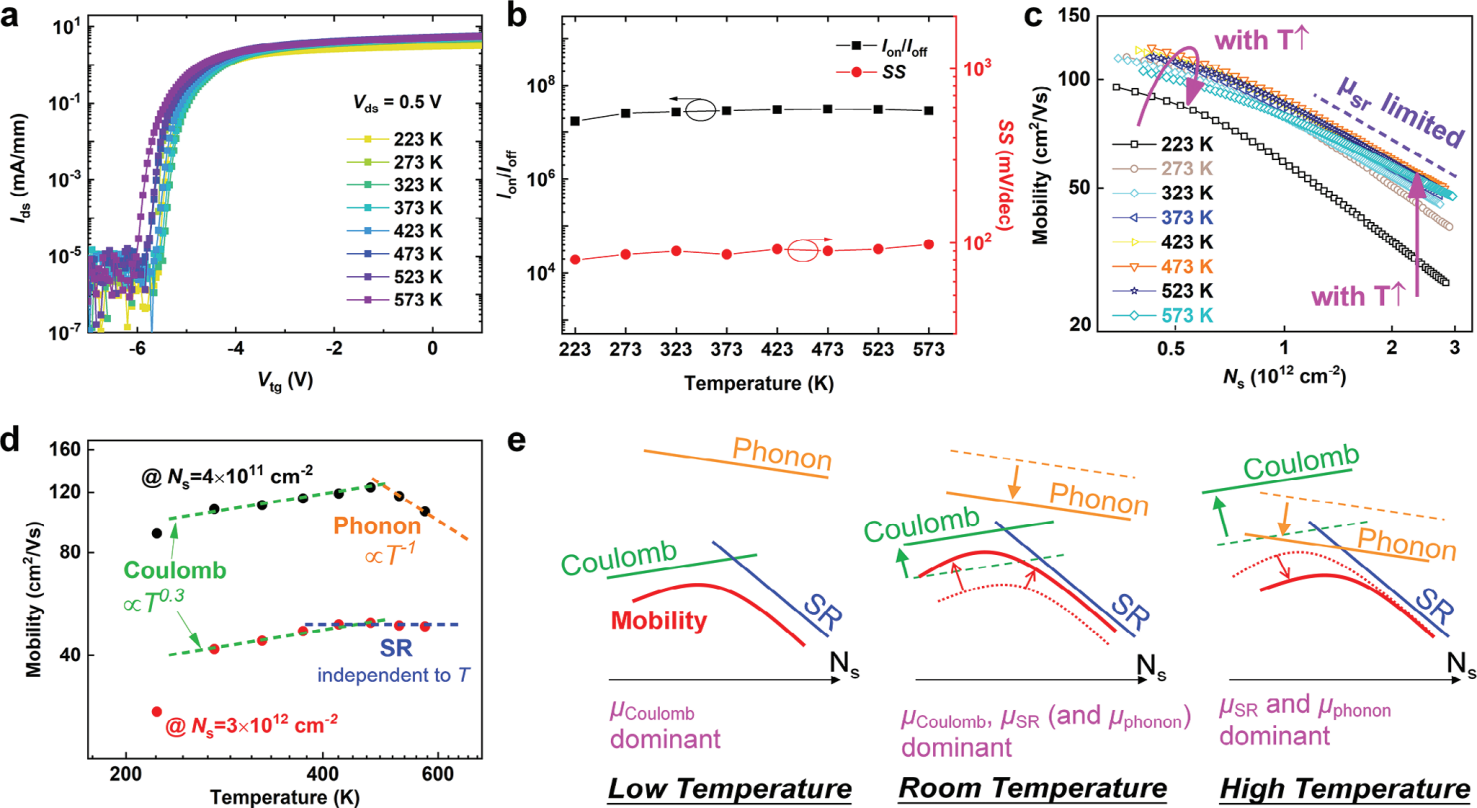
a) The temperature‐dependence transfer curves of h‐BN/β‐Ga_2_O_3_ HJFET in log‐scale from 223 to 523 K at Vds = 0.5 V. b) Changes of extracted Ion/Ioff and SS as a function of temperature. c) The low and high‐temperature mobility in the h‐BN/β‐Ga_2_O_3_ HJFET. d) The temperature dependence of low and high Ns mobility in h‐BN/β‐Ga_2_O_3_ HJFET. e) The carrier scattering mechanisms in the h‐BN/β‐Ga_2_O_3_ HJFET at different temperature regions.

As is well known, the thermal stability and reliability of the electrical device are very important in practical applications.^[^ ^9^, ^45^ ^]^ To explore the thermal reliability of the presented h‐BN/*β*‐Ga_2_O_3_ HJFET, we performed temperature‐dependent electrical tests at a wide temperature range from 223 to 573 K. These measurements were performed using a high‐low temperature probe station equipped with a heating system, with a heating rate of ≈5 °C min^−1^. At each target temperature, the device was held for 10 min to achieve thermal equilibrium before recording its electrical characteristics. The temperature‐dependence transfer curves of h‐BN/*β*‐Ga_2_O_3_ HJFET in log‐scale at *V*_ds_ = 0.5 V are shown in **Figure** **2**a. It can be found that the h‐BN/*β*‐Ga_2_O_3_ HJFET can maintain a great gate control characteristic in a wide temperature range. However, the off‐state current exhibits fluctuations during temperature‐dependent testing. At higher temperatures, the thermal activation of carriers becomes significant, while at lower temperatures, charge trapping and de‐trapping processes may dominate.^[^ ^46^ ^]^ Then, from the transfer curves we plotted the curves of *I*_on_/*I*_off_ and *SS* as a function of temperature in Figure 2b. Obviously, the high *I*_on_/*I*_off_ and steep *SS* remain stable throughout the temperature change. Note that, after completing the high and low‐temperature tests, we tested the electrical characteristics of the device back to room temperature immediately, as shown in Figures S7–S9 (Supporting Information). The experimental results indicate that the electrical characteristics of the device could be restored to its original characteristics before variable the temperature test. According to the above results, it can be concluded that the h‐BN/*β*‐Ga_2_O_3_ HJFET has a high‐temperature stability. To comprehensively understand the temperature‐dependent electrical properties of the h‐BN/*β*‐Ga_2_O_3_ HJFET, the mobility in the *β*‐Ga_2_O_3_ channel is further evaluated at different temperature conditions. Figure 2c shows the relationship between mobility and surface carrier concentration (*N*_S_) which is defined by *Q_i_*/*q*, where *q* is electron charge.^[^ ^35^ ^]^ Here, As summarized in Figure 2d, the mobility first increases in both low *N*s (4 × 10^11^ cm^−3^) and high *N*s (3 × 10^12^ cm^−3^) regions with a higher temperature and then achieves the maximum value at 473 K. This phenomenon is attributable to the physical origin that the mobility under 473 K is limited by Coulomb scattering.^[^ ^47^ ^]^ With further increase of temperature, the high *N*s mobility achieves a maximum level and do not change with temperature, suggesting that the high *N*s mobility above 473 K is limited by surface roughness scattering.^[^ ^48^ ^]^ The low *N*s mobility at >473 K decreases with increasing temperature, attributable to the increase of phonon scattering.^[^ ^48^ ^]^ To be more detailed, the carrier scattering mechanisms in the *β*‐Ga_2_O_3_ channel are summarized in Figure 2e. The mobility in *β*‐Ga_2_O_3_ channels is limited by Coulomb scattering at low‐temperature (< 473 K) and limited by phonon and surface roughness scattering at high‐temperature (> 473 K). These results also indicate that the electrical performance of the h‐BN/*β*‐Ga_2_O_3_ HJFETs can be further boosted through suppression of Coulomb scattering centers at the MIS interface and reduction of MIS interface roughness.

**Figure 3:**
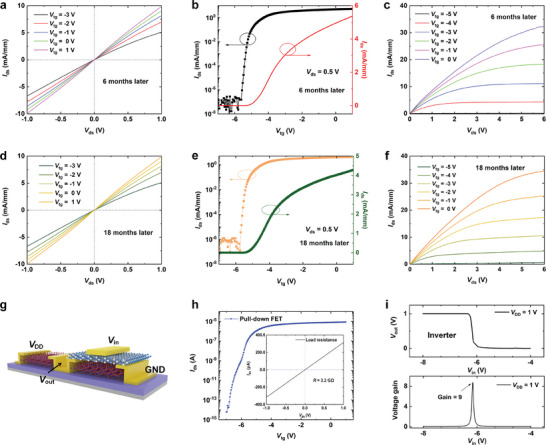
a–c) The electrical characteristics of h‐BN/β‐Ga_2_O_3_ HJFET after being placed in an air ambient for 6 months. a) Ohmic contact characteristics of the device under different Vtg values. b) The transfer curves of h‐BN/β‐Ga_2_O_3_ HJFET in the log‐ and linear‐ scales. c) The output curves of h‐BN/β‐Ga_2_O_3_ HJFET. d–f), The electrical characteristics of h‐BN/β‐Ga_2_O_3_ HJFET after being placed in an air ambient for 18 months. d) Ohmic contact characteristics of the device under different Vtg values. e) The transfer curves of h‐BN/β‐Ga_2_O_3_ HJFET in the log‐ and linear‐ scales. f) The output curves of h‐BN/β‐Ga_2_O_3_ HJFET. g) Schematic diagram of the inverter based on h‐BN/β‐Ga_2_O_3_ HJFET. h) The transfer curve of the pull‐down FET and the output curve of Rload. i) Top, Dependence of output voltage of the inverter on input voltage at VDD = 1 V. Bottom, the voltage gain of the inverter at VDD = 1 V.

Furthermore, the long‐term environmental stability of the h‐BN/*β*‐Ga_2_O_3_ HJFET was investigated which is another major challenge for its practical application and commercialization.^[^ ^49^ ^]^ The device is placed in an air ambient for 18 months, and then we tested its electrical characteristics every 6 months. **Figure** **3**a–c shows the Ohmic contact properties, transfer curves, and transport curves of the device after storage for 6 months. No degradation was observed, which indicates the high stability of the device based on h‐BN/*β*‐Ga_2_O_3_ in an air ambient. Compared to other 2D materials such as transition‐metal dichalcogenides, *β*‐Ga_2_O_3_ materials has a higher stability to resist humid environments.^[^ ^50^, ^51^ ^]^ Next, the electrical properties of fabricated *β*‐Ga_2_O_3_ HJFET after storage for 12 and 18 months are shown in Figures S10–S12 (Supporting Information) and Figure 3d–f, respectively. It can be found that even if the device is placed in an air ambient for a full 18 months, it still maintains reliable electrical properties, showing excellent contact characteristics, a great gate‐controlled switching capability, and a large saturated output current. The results indicate that *β*‐Ga_2_O_3_‐based devices have long‐term environmental stability even after the variable temperature measurements.

Based on the advantages of the above‐mentioned h‐BN/*β*‐Ga_2_O_3_ HJFET, it is expected for the practical application in logic circuits by manufacturing representative logic inverters. Figure 3g shows the schematic diagram of the logic inverter structure. The optical micrograph of the fabricated inverter device is shown in Figure S13 (Supporting Information). The inverter logic circuit was constructed by connecting a h‐BN/*β*‐Ga_2_O_3_ HJFET and a load resistance (*R*_load_). The transfer curve of the pull‐down h‐BN/*β*‐Ga_2_O_3_ HJFET and the output curve of *R*_load_ are displayed in Figure 3h. The h‐BN/*β*‐Ga_2_O_3_ HJFET exhibits good electrical properties with low *I*_off_ of ≈10 fA and high *I*_on_/*I*_off_ of ≈10^8^ and the value of *R*_load_ is calculated to be 3.2 GΩ. The top Figure in Figure 3i plots the voltage transfer characteristic of the logic inverter at supply voltage *V*_DD_ = 1 V. The switching voltage is measured to be −6.15 V. Then, the voltage gain (*V*_gain_) showing the sensitivity of *V*_out_ switching to *V*_in_ is also extracted by using the equation of *V*_gain_ = −d*V*_out_/d*V*_in_ in the bottom Figure of Figure 3i. It can be seen that the voltage gain reaches a large value of 9 at *V*_DD_ = 1 V, indicating the capability of the fabricated n‐logic inverter to be used as a multilevel circuit building block. Meanwhile, the dynamic switching characteristic of the inverter is captured with *V*_DD_ = 1 V at a frequency of 1 Hz in Figure S14 (Supporting Information). The output curve is opposite to the input curve and no significant delay is observed.

**Figure 4:**
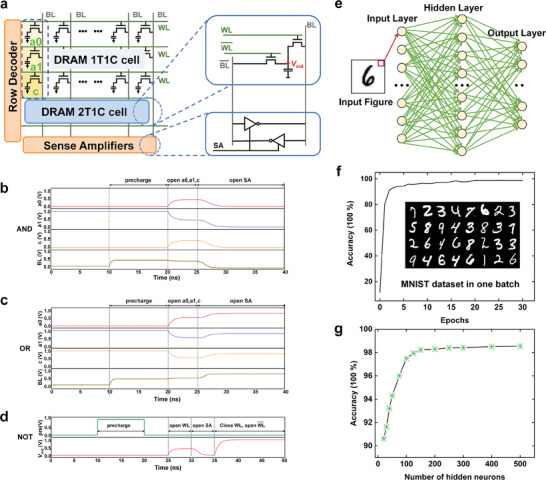
a) The basic structure of the DRAM memory calculation circuit, including the DRAM 1T1C cell array, a row of 2T1C units, row decoders and sensitive amplifier circuits, and other peripheral circuits. On the right is a specific structure of a 2T1C unit. b–d), AND, OR, and NOT operation waveforms based on h‐BN/β‐Ga_2_O_3_ HJFET, respectively. e) Illustration diagram of fully connected neural network structure. f) The recognition rates of the neural network of MNIST image recognition. g) The relationship between the number of hidden layer neurons and the accuracy of MNIST image recognition.

Next, due to the great long‐term environmental stability and temperature reliability in a wide range of the proposed h‐BN/*β*‐Ga_2_O_3_ HJFET, it is further expected to extend to the potential applications in memory and artificial intelligence fields in harsh environment. As is well known, the computing‐in‐memory accelerator is crucial for improving the operating speed of the equipment. First, a DRAM structure based on h‐BN/*β*‐Ga_2_O_3_ HJFET was proposed and successfully implemented as a computing in‐memory accelerator.^[^ ^52^ ^]^ **Figure** **4**a shows the basic structure of computing in‐memory circuits based on DRAM, including DRAM one‐transistor‐one‐capacitance (1T1C) cell arrays, a row of two‐transistors‐one‐capacitance (2T1C) units, row decoders, sensitive amplifier (SA) circuits, and other peripheral circuits. In this work, we simultaneously activated three rows (such as *a0*, *a1*, *c* cells) and controlled the initial value of one of the three rows to achieve a bitwise AND or OR logic operation of the other two rows. In other words, if *A*, *B*, and *C* represent the logical values of the three cells, the final state of the bit line (BL) is *AB* + *BC* + *CA*, that is, *C*(*A* + *B*) + $\bar{C}$(*AB*). So the bitwise AND or bitwise OR of *a0* and *a1* cells can be achieved by controlling the value of *c* cell. Figure 4b,c shows the AND and OR operation waveforms of DRAM, respectively. Here, precharge is the precharge stage which makes the voltage on BL reach *V*_DD_/2, open *a0*/*a1* refers to opening the word line (WL) of the corresponding DRAM cell, and open SA means to enable the sensitive amplifier. The *a0* cell capacitor is initially empty and the *a1* cell capacitor is initially fully charged. Obviously, when the *c* cell is with a logical value of 0, the AND operation will be performed. To be detailed, after open *a0*, *a1*, and *c* cells, they would cause a negative deviation on the BL due to charge sharing. Then the SA drives the BL to 0 V, and as a result, fully empty all the three cells. The AND operation has been performed when the final state of the BL becomes 0, as shown in Figure 4b. And it can be deduced that when the *c* cell is with logical value of 1, the OR operation will be performed, and the final state of the BL outputs 1, as shown in Figure 4c. Subsequently, a row of 2T1C cell is connected to both sides of the inverter to execute bitwise NOT of any row of DRAM cells. The enlarged 2T1C unit is displayed in the right side of Figure 4a. Figure 4d shows the NOT operation waveforms of *β*‐Ga_2_O_3_‐based DRAM. Assuming the cell capacitor is initially empty, open the WL, and the value of the BL becomes lower than the $\bar{\mathrm{BL}}$ due to the charging of the capacitor. Then the SA drives the BL to 0 V and $\bar{\mathrm{BL}}$ to 1 V. Finally, open the $\bar{\mathrm{WL}}$ and close the WL, the capacitor is finally fully charged, and a high‐level logic 1 is output. Visibly, the DRAM memory calculation circuit based on *β*‐Ga_2_O_3_ device could efficiently perform AND, OR, and NOT operations. Therefore, the in‐memory calculation module can perform the efficient multiply‐accumulate operations, which are the main calculations used in artificial intelligence.^[^ ^53^ ^]^ Besides, a fully connected network (FNN) model consisting of 3 layers is considered for a handwritten digit recognition task based on the MNIST dataset. The MNIST dataset consists of 60 000 training images and 10 000 testing images and the Backpropagation algorithm is used to train the weights of the synapses. As shown in Figure 4e, the 3‐layer FNN consists of the input layer, the hidden layer, and the output layer. And the 784 input neurons correspond to the 28 × 28 pixels in an image and 10 output neurons correspond to the recognition of numbers 0–9, respectively. In such a neuron network, each neuron receives inputs from all neurons in the previous layer. Figure 4f shows the recognition rates of the FNN of MNIST image recognition. After five training epochs, the corresponding recognition rate could reach 95%, which exhibits high image recognition efficiency. But the number of hidden layers also greatly affects the speed and accuracy of neural network operations. Next, we changed the number of hidden layers to verify its impact on the accuracy of the neural network. As shown in Figure 4g. It can be found that the more hidden layers of the neural network, the higher the accuracy of its recognition. All results demonstrate that h‐BN/*β*‐Ga_2_O_3_ HJFET exhibits great potential in emerging fields such as artificial intelligence and image recognition fields.

## 3 Conclusion

In summary, a h‐BN/*β*‐Ga_2_O_3_ HJFET has been fabricated with a low *I*_off_ of ≈10 fA, a high *I*_on_/*I*_off_ of ≈10^8^, a low *R*_c_ of 5.6 Ω·mm, and a high μ_fe_ of 156 cm^2^ (Vs)^−1^. Importantly, the obtained h‐BN/*β*‐Ga_2_O_3_ HJFET shows not only good thermal reliability in a temperature range of 223–573 K but also high environmental stability even after 18 months. Besides, the h‐BN/*β*‐Ga_2_O_3_ HJFET can also be used successfully in accelerator‐in‐memory computing fields including DRAM and neural network computation applications. This work provides a new perspective for the development of next‐generation nanoelectronics.

## 4 Experimental Section

### Device Fabrication

In this work, the *β*‐Ga_2_O_3_ bulk single crystal with 0.05 mol % Ta doping was used. It exhibits an active electron concentration of 1.4 × 10^18^ cm^−3^ measured by Hall effect and electrical resistivity tests. Subsequently, *β*‐Ga_2_O_3_ flakes were exfoliated mechanically from *β*‐Ga_2_O_3_ bulk by commercial Scotch tape and transferred onto 110 nm SiO_2_/p^++^‐Si substrate. Then, the electron‐beam lithography (EBL) process was used to pattern the source/drain regions, followed by the development, e‐beam evaporation, and lift‐off processes. The stack metal of Ti/Al/Ni/Au (20/100/60/80 nm) was chosen to use as a source/drain electrode. And an RTA process in a high purity N_2_ ambient was also carried out at 470 °C for 1 min to improve electrode/channel contact. After the fabrication of a two‐terminal *β*‐Ga_2_O_3_ device, the mechanically exfoliated h‐BN flake was precisely transferred to the top of the target *β*‐Ga_2_O_3_ channel as the gate dielectric layer using a poly (dimethylsiloxane) transfer method. Finally, the top gate composed of Ti/Au (10/70 nm) was defined via a second EBL, metallization, and lift‐off processes to finish the fabrication of h‐BN/*β*‐Ga_2_O_3_ HJFET.

### Microscopic and Electrical Characterizations

The crystalline structure of *β*‐Ga_2_O_3_ bulk single crystal was obtained by the Synchrotron‐based GIXRD which uses X‐ray with a wavelength of 0.6887 Å. The Raman spectrum was investigated using a commercial Raman spectrometer (Horiba Jobin Yvon LabRAM HR800) equipped with an excitation wavelength of 532 nm. The *β*‐Ga_2_O_3_ flakes structural analysis was analyzed using HR‐TEM (Talos F200X). The thicknesses of h‐BN and *β*‐Ga_2_O_3_ flakes were determined via AFM (Bruker Dimension Icon). The all‐electrical measurements were conducted by using a semiconductor analyzer (Agilent B1500A).

## Conflict of Interest

The authors declare no conflict of interest.

## Supporting information



Supporting Information

## Data Availability

The data that support the findings of this study are available from the corresponding author upon reasonable request.
