# Supplementary material for: Highly Stable Electronics Based on β‐Ga2O3 for Advanced Memory Applications
Source: Adv Sci (Weinh). 2025 Feb 5;12(12):2413846. doi: 10.1002/advs.202413846 (PMC11948083; doi:10.1002/advs.202413846)
Supplement: Supplementary file 1 — Supporting Information [file ADVS-12-2413846-s001.docx]

Supporting information

**Highly stable electronics based on *β*-Ga_2_O_3_ for advanced memory applications**

Xiao-Xi Li^1,2^, Yu-Chun Li^3^, Yingguo Yang^3,4,5*^, Bitao Dong^6,7^, Yuhang Liu^6^, Lina Li^4^, Linfeng Pan^8*^, Gengsheng Chen^3,*^, Yue Hao^1,2^, and Genquan Han^1,2*^

^1^Hangzhou Institute of Technology, Xidian University, Hangzhou, 311200, China

^2^School of Microelectronics, Xidian University, Xi’an, 710071, China

^3^State Key Laboratory of ASIC and System, Shanghai Institute of Intelligent Electronics & Systems, School of Microelectronics, Fudan University, Shanghai 200433, China

^4^Shanghai Synchrotron Radiation Facility (SSRF), Zhangjiang Lab, Shanghai Advanced Research Institute, Chinese Academy of Sciences, Shanghai 201204, China.

^5^State Key Laboratory of Photovoltaic Science and Technology, Fudan University, Shanghai 200433, China.

^6^State Key Laboratory for Mechanical Behavior of Materials, School of Materials Science and Engineering, Xi’an Jiaotong University, Xi’an, 710049, China

^7^Department of Materials Sciences and Engineering, Division of Solid State Physics, Angstrom Laboratory, Uppsala University, Uppsala, SE-75105, Sweden

^8^Department of Chemical Engineering and Biotechnology, University of Cambridge, Cambridge, UK.

*Email: [yangyingguo@fudan.edu.cn,](mailto:yangyinguo@fudan.edu.cn,) [lp577@cam.ac.uk,](mailto:lp577@cam.ac.uk,) gschen@fudan.edu.cn, [gqhan@xidian.edu.cn](mailto:gqhan@xidian.edu.cn)

*Density Functional Theory (DFT) Calculations*

First-principles calculations of this work were performed which based on the DFT+U framework. The projector augmented wave method and the Perdew-Burke-Ernzerhof functional were selected, as conducted in the Vienna *ab-initio* simulation package. Ga s2p1 and O s2p4 are considered as valence electrons. For the heterostructure simulation, we use a $4\times\sqrt{3}$ superlattice for h-BN monolayer on a $2\times3$ superlattice for Ga_2_O_3_ surface for lattice matching. The h-BN and Ga_2_O_3_ unit cells are optimized using a Monkhorst-Pack k-point meshes of 21×21×1 and 6×4×1 separately. The 136-atom heterostructure SCF calculation is conducted using Γ point only. The kinetic energy cut-off for plane-wave basis set was set to 450 eV. The geometry optimizations were analyzed until the Hellmann-Feynman force affecting per atom was less than 1×10^−4^ eV/Å. The energy convergence criterion was set to 1×10^−7^ eV.


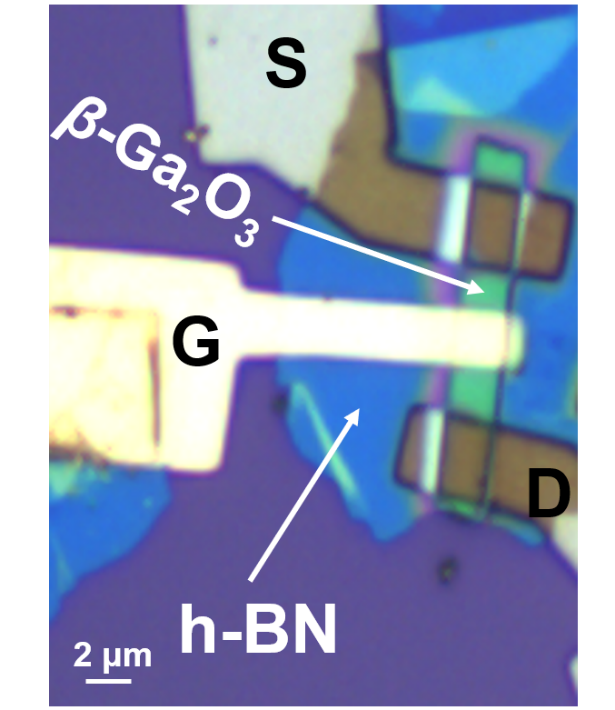


**Figure S1** The optical microscopic image of the fabricated h-BN/*β*-Ga_2_O_3_ HJFET.


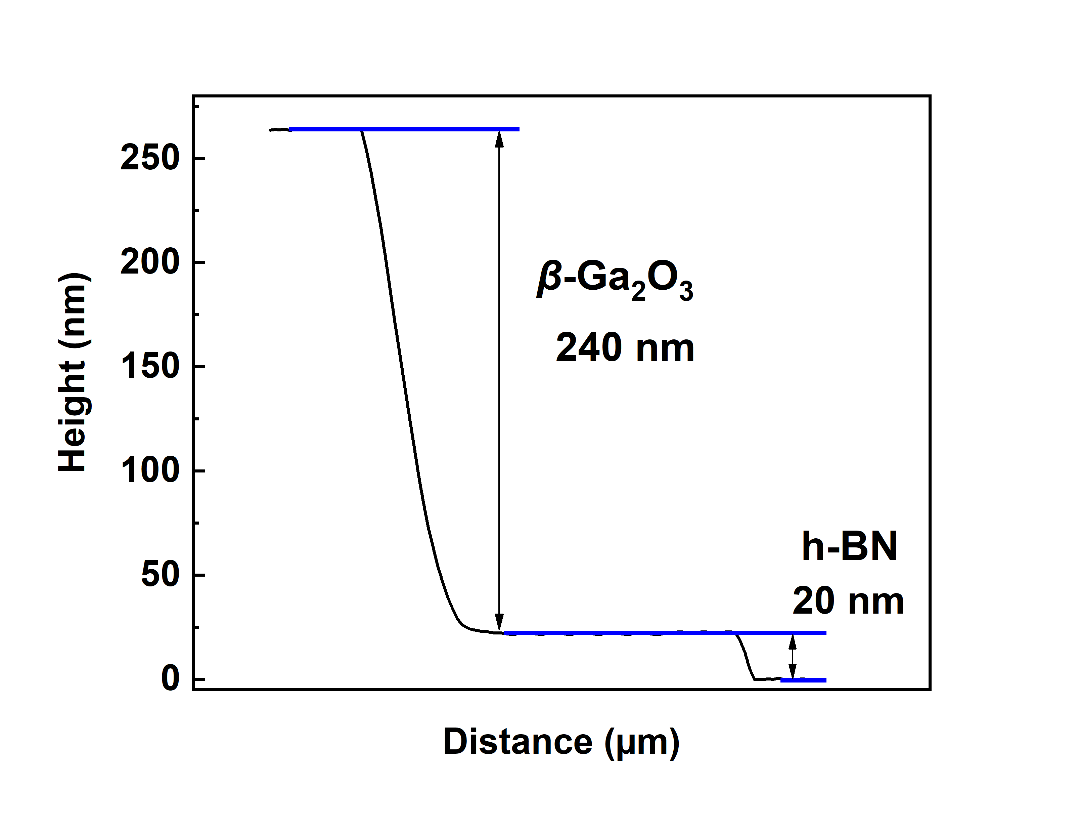


**Figure S2** Height profile of the exfoliated h-BN and *β*-Ga_2_O_3_ flakes.


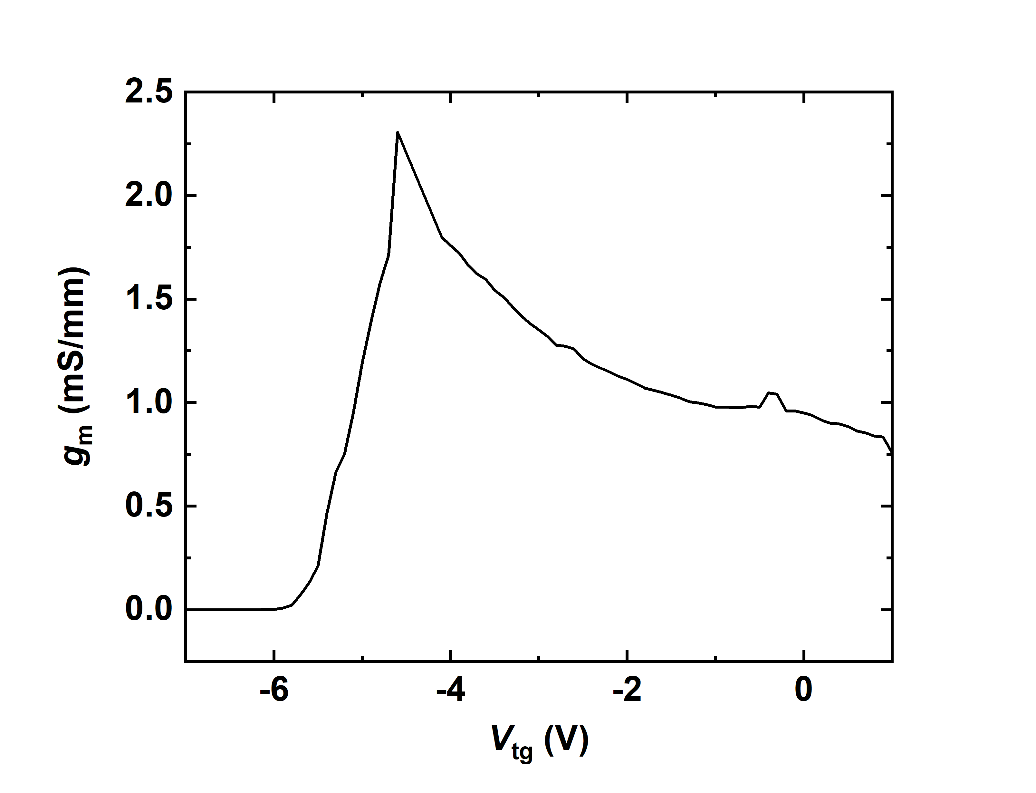


**Figure S3** The transconductance as a function of *V*_tg_.


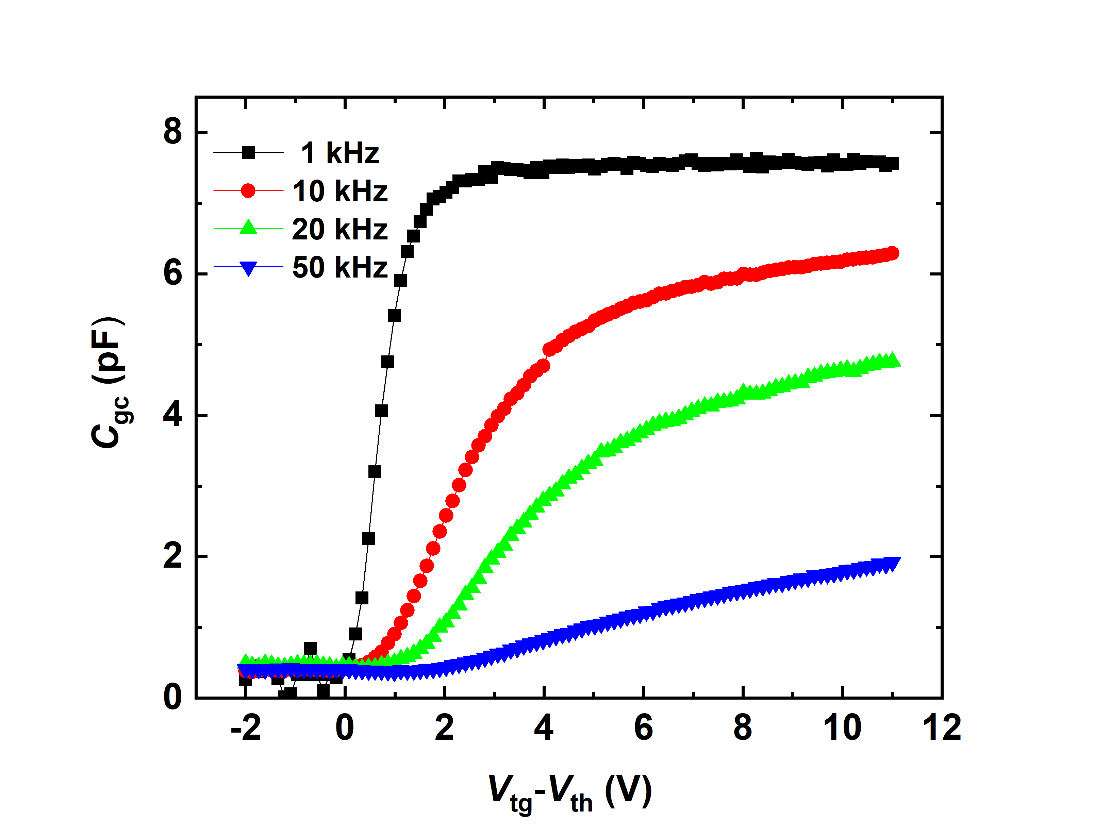


**Figure S4** The *C*_gc_-*V*_tg_ curves taken from a h-BN/*β*-Ga_2_O_3_ structure.


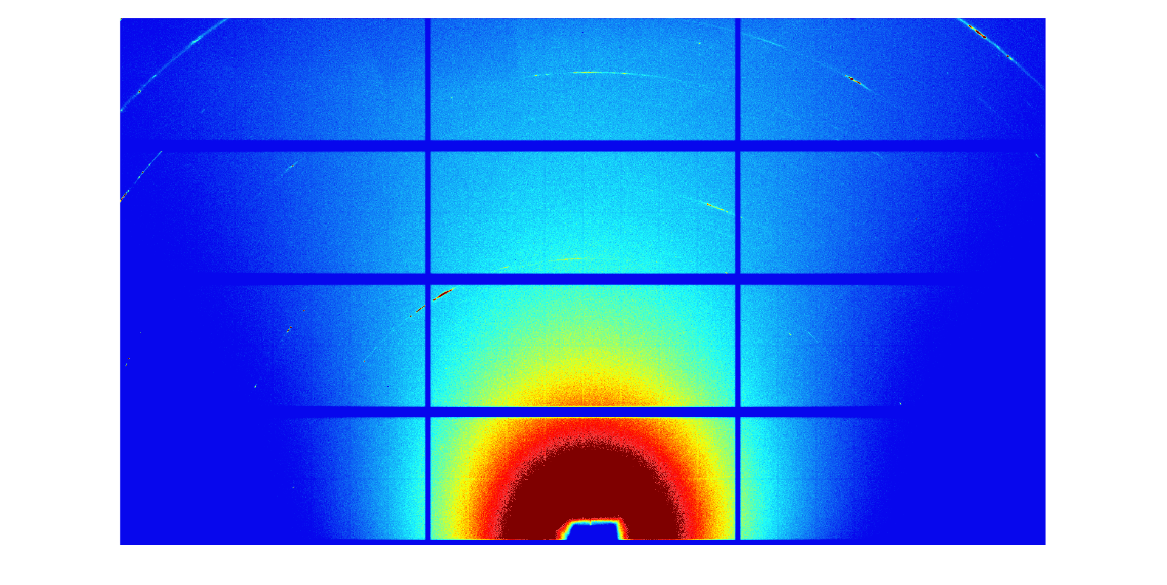


**Figure S5** two-dimensional grazing incident X-Ray diffraction pattern of the *β*-Ga_2_O_3_ sample.


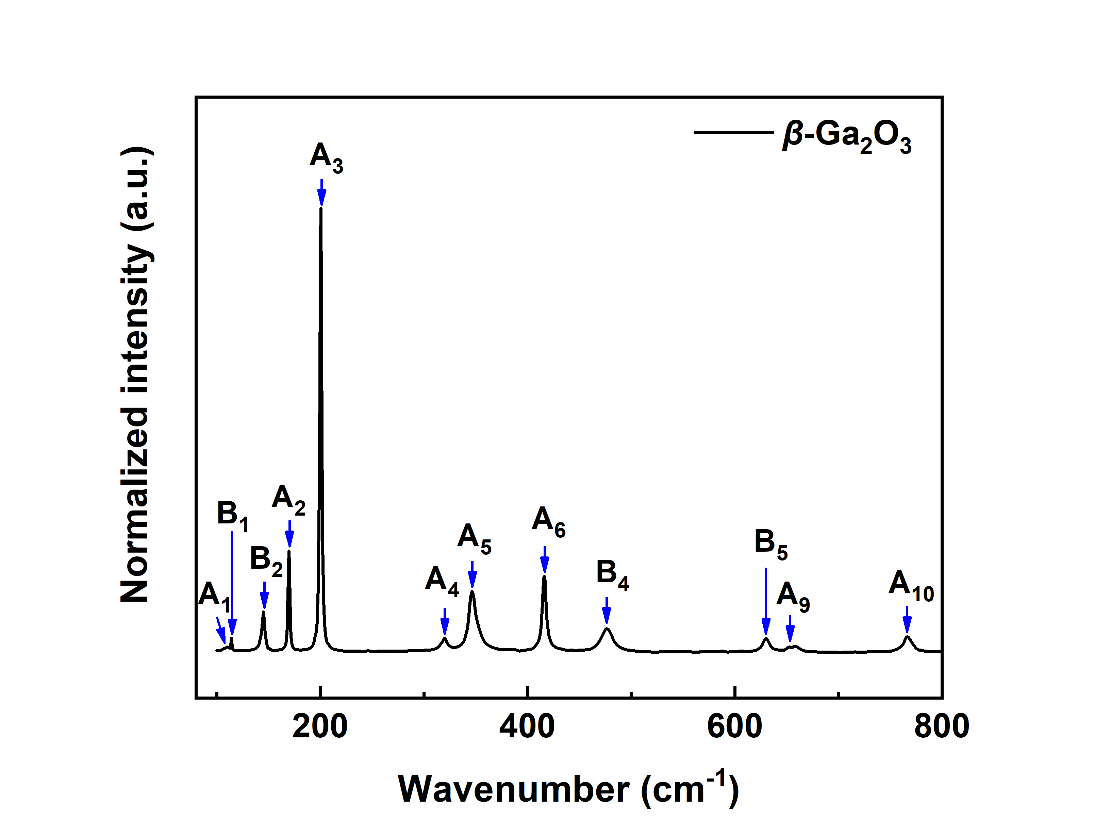


**Figure S6** Raman spectrum of *ꞵ*-Ga_2_O_3_ bulk single crystal.


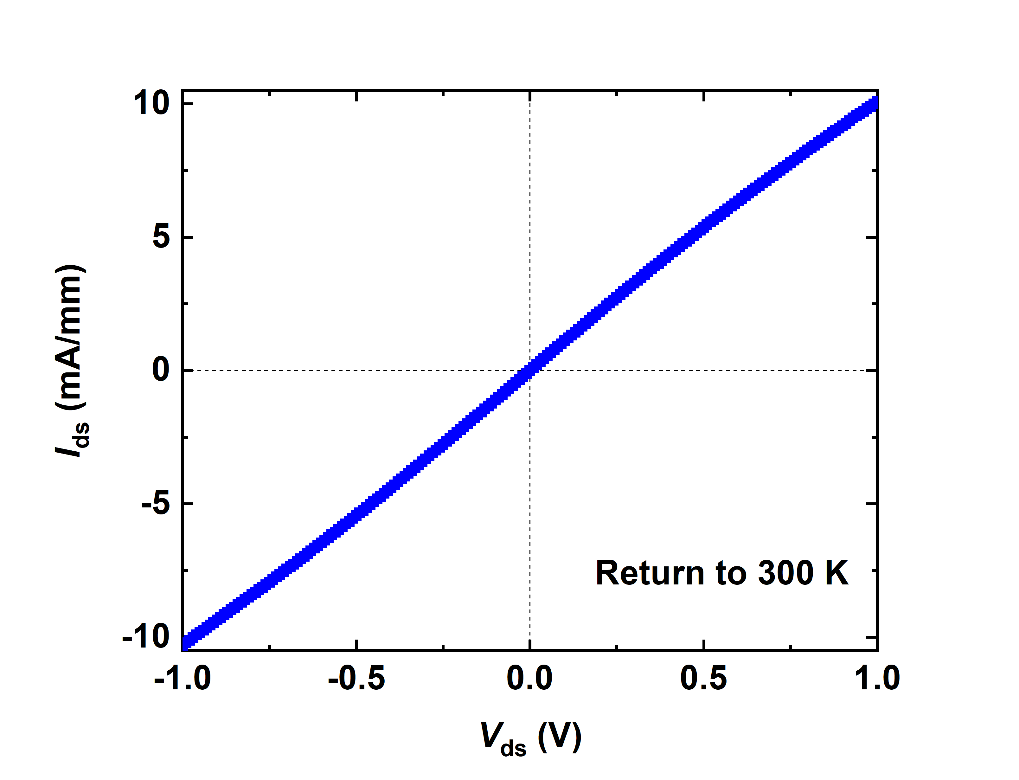


**Figure S7** Ohmic contact characteristics of h-BN/*β*-Ga_2_O_3_ HJFET after returning to room temperature 300 K.


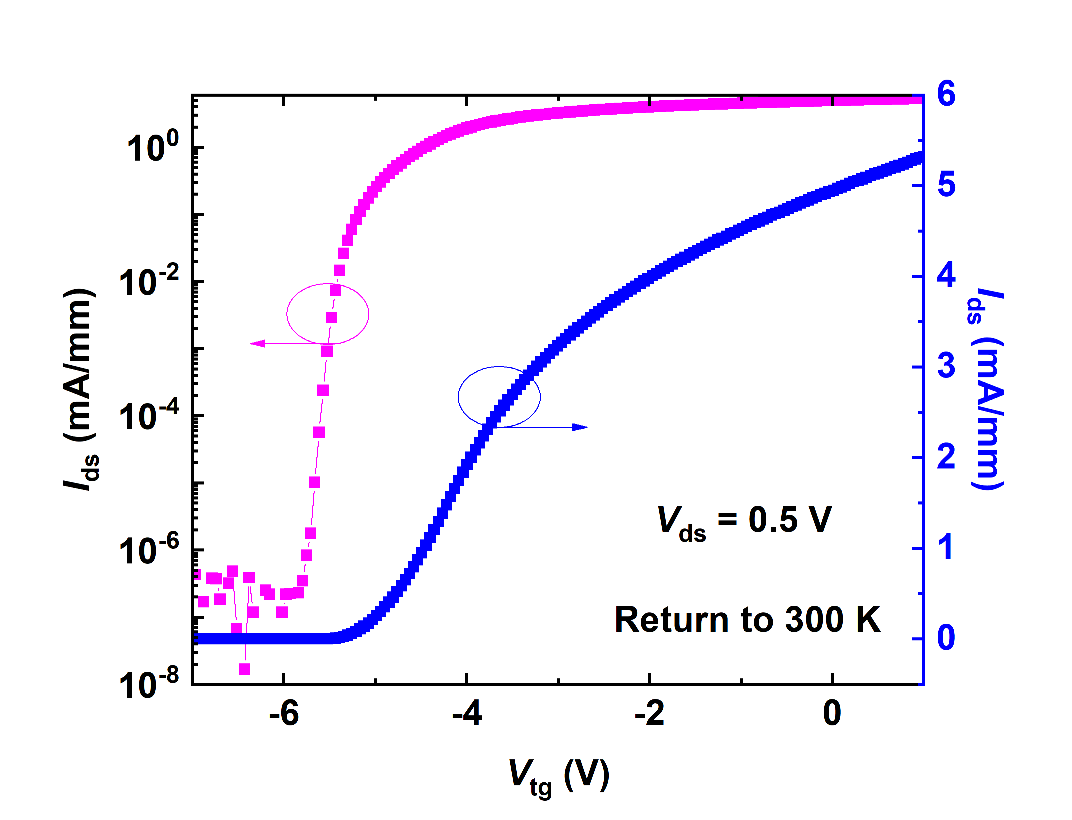


**Figure S8** The transfer curves of h-BN/*β*-Ga_2_O_3_ HJFET in the log- and linear- scales after returning to room temperature 300 K.


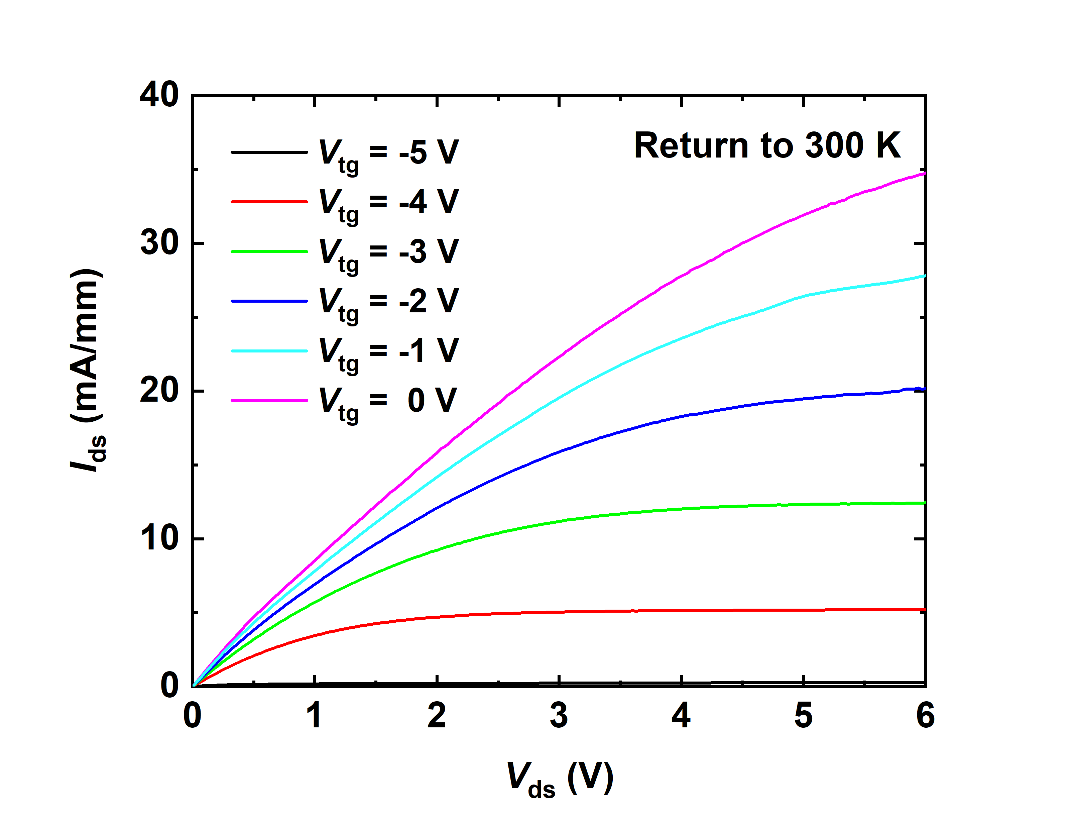


**Figure S9** The output curves of h-BN/*β*-Ga_2_O_3_ HJFET after returning to room temperature 300 K.


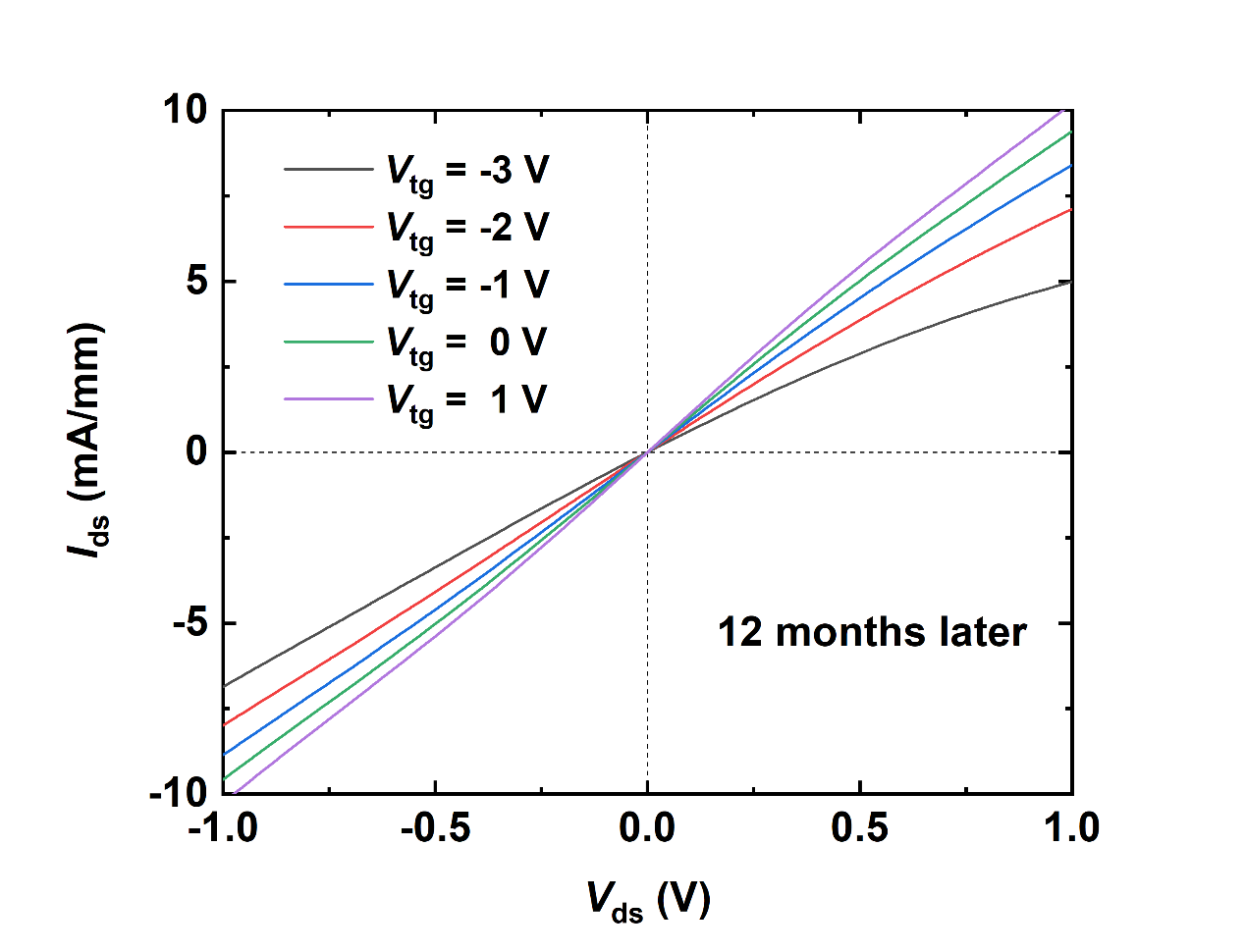


**Figure S10** Ohmic contact characteristics of h-BN/*β*-Ga_2_O_3_ HJFET after being placed in air for 12 months.


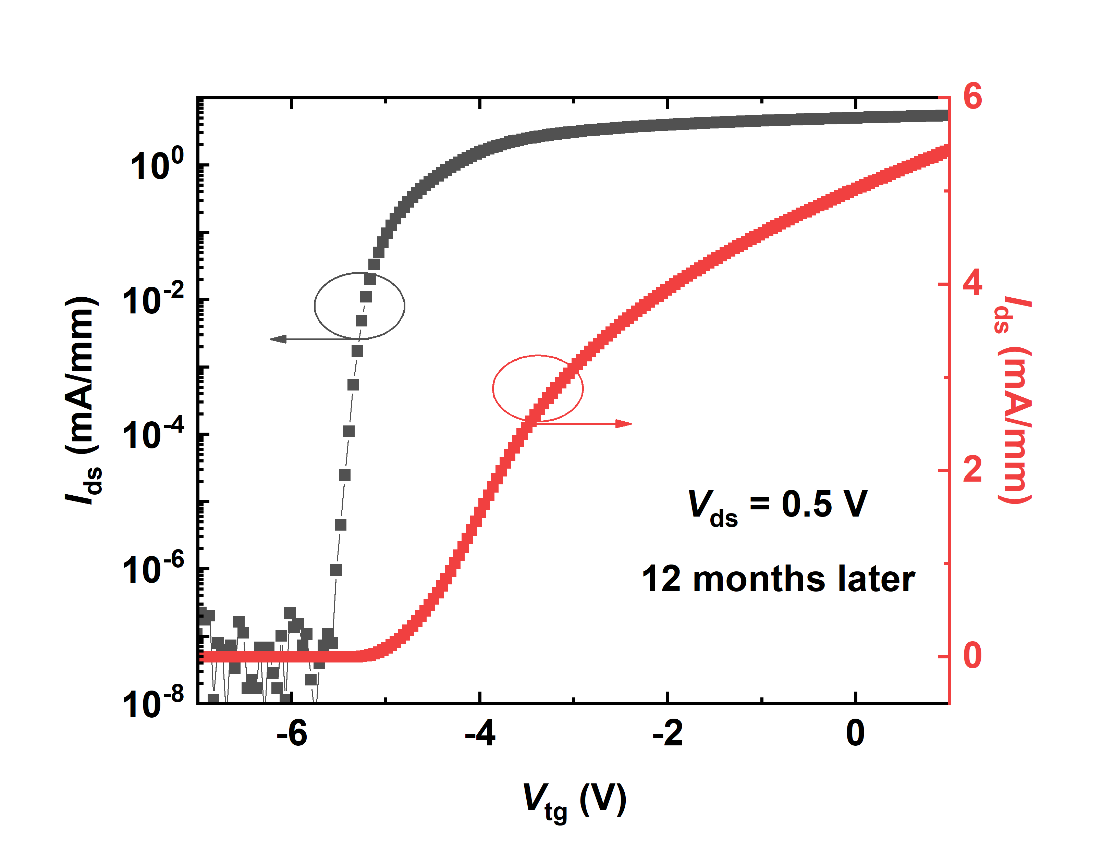


**Figure S11** The transfer curves of h-BN/*β*-Ga_2_O_3_ HJFET in the log and linear scale after being placed in air for 12 months.


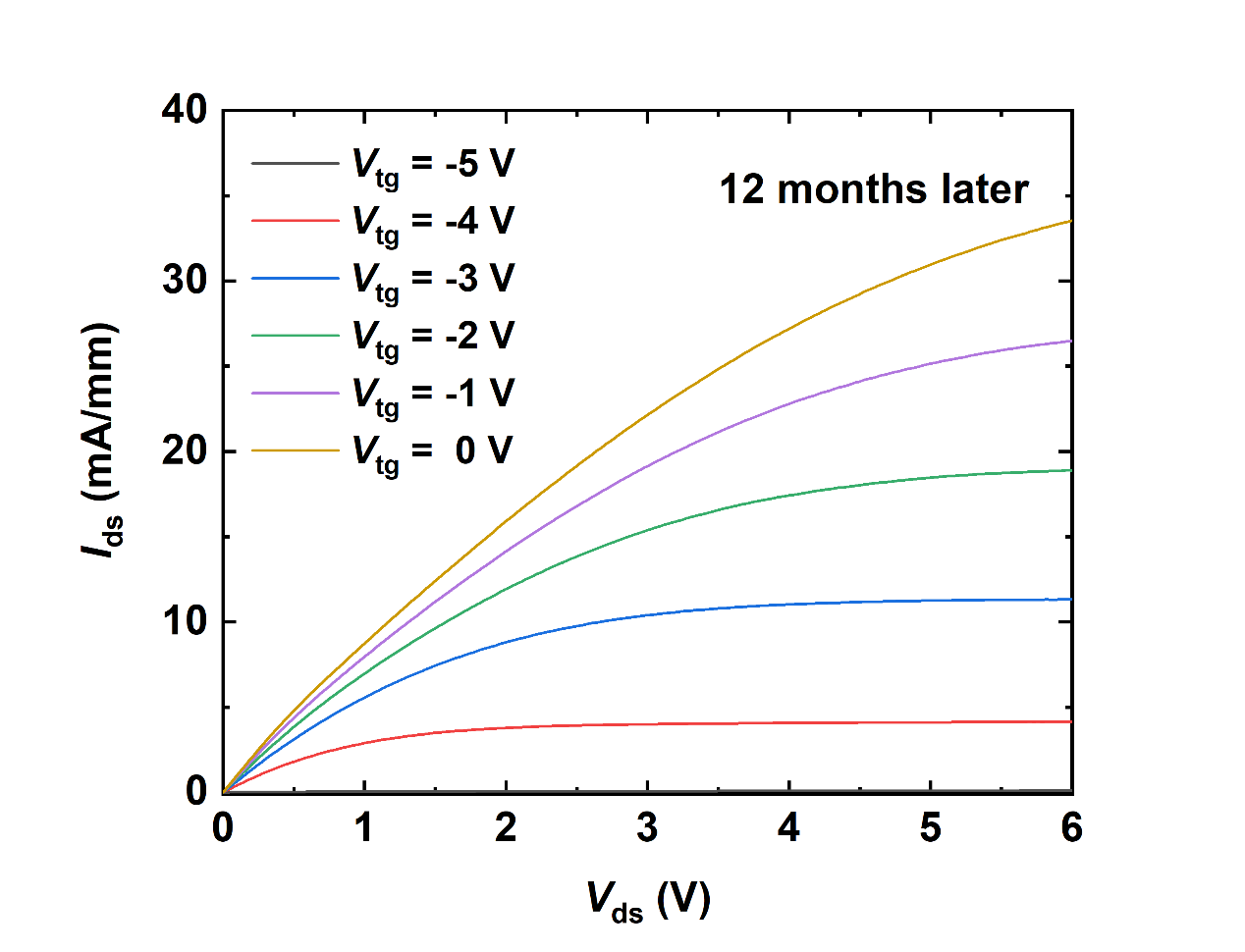


**Figure S12** The output curves of h-BN/*β*-Ga_2_O_3_ HJFET after being placed in air for 12 months.


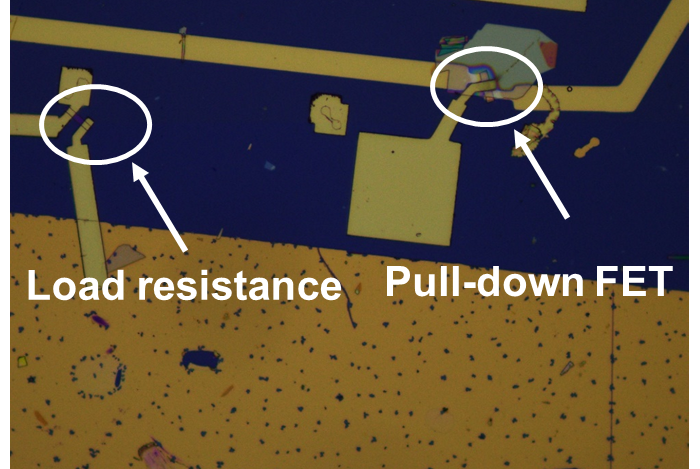


**Figure S13** The optical image of the inverter based on h-BN/*β*-Ga_2_O_3_ HJFET.


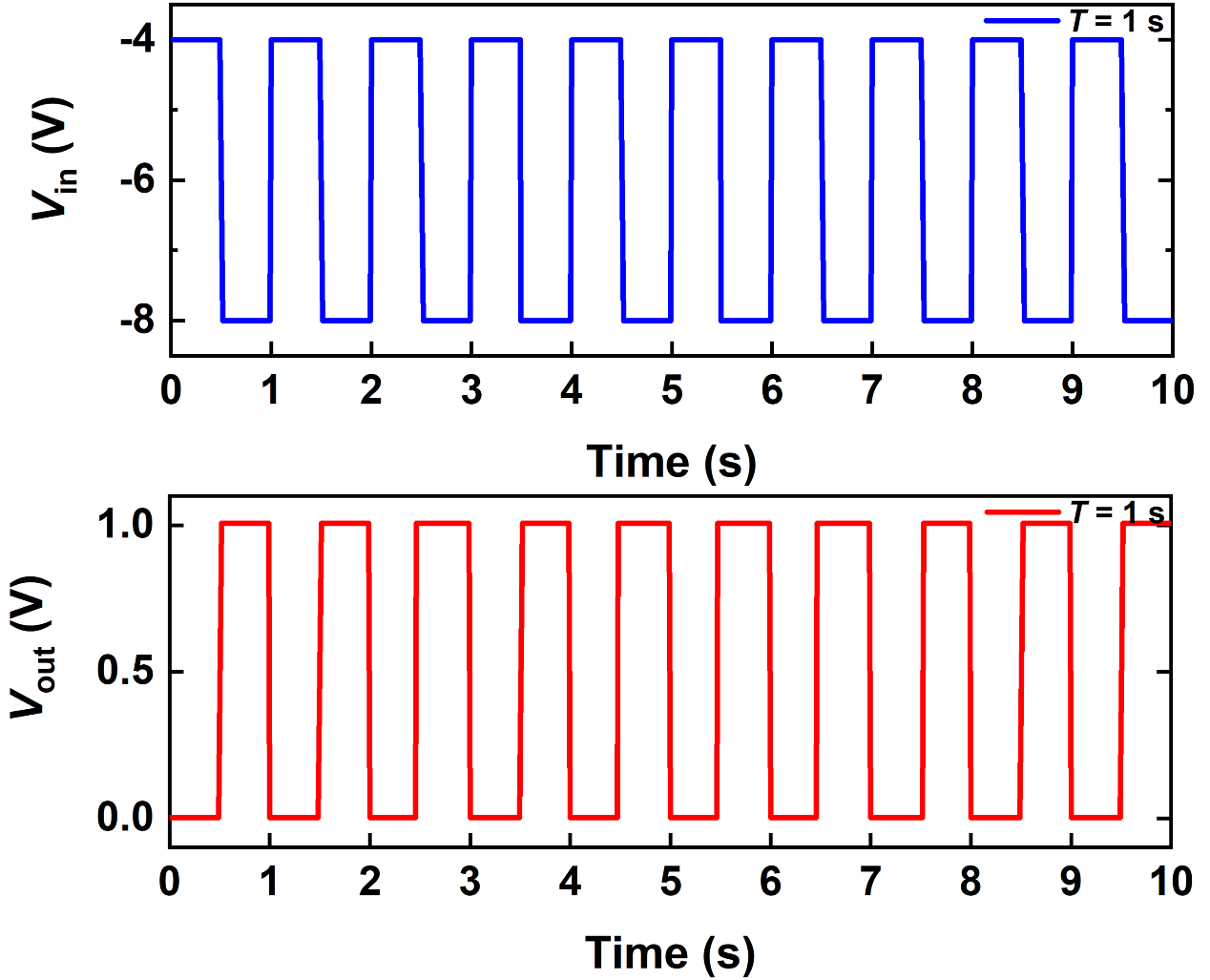


**Figure S14** Dynamic switching characteristic of inverter at a frequency of 1 Hz.
